# Comparative diagnostic accuracy of multiple large language models in oral and maxillofacial radiology specialty examinations: a 13-year analysis of performance and topic trends

**DOI:** 10.1186/s12903-026-08236-3

**Published:** 2026-03-31

**Authors:** Murat Icen, Katibe Tugce Temur

**Affiliations:** 1 Department of Oral and Maxillofacial Radiology, Faculty of Dentistry, Nevşehir Hacı Bektaş Veli University, Nevşehir, Türkiye; 2 Department of Oral and Maxillofacial Radiology, Faculty of Dentistry, Niğde Ömer Halisdemir University, Niğde, Türkiye

**Keywords:** Oral and maxillofacial radiology, Dental specialty examination, Large language models, Artificial intelligence, Educational assessment

## Abstract

**Background:**

To the best of our knowledge, this is the first study in oral and maxillofacial radiology to comprehensively include all examination questions, to systematically analyse their topic distribution across years and examination periods, and to concurrently compare multiple contemporary large language models within a unified methodological framework. This study aimed to compare the accuracy performance of six different artificial intelligence (AI) systems based on large language models (LLMs) of questions asked in the field of oral and maxillofacial radiology in the Dental Specialization Examination (DSE) over the past 13 years, and to analyze the subject matter in detail.

**Methods:**

A total of 200 oral and maxillofacial radiology questions from the DSE held between 2012 and 2025 were included in the analysis. The questions were grouped according to their topics and divided into early-late periods (2012–2018 and 2019–2025) to observe changes over time. ChatGPT-5.2, ChatGPT-4.0, Gemini-3, Claude 4.5, Microsoft Copilot, and Perplexity AI were tested using the original question formats. The models’ answers were evaluated against the official answer key. In addition, the questions were analyzed according to exam years and periods.

**Results:**

In the early and late periods, ChatGPT-5.2 showed accuracy rates of 91.9% and 95.7%, respectively. This was followed by ChatGPT-4.0 (79.8% − 82.8%). Differences between the models were statistically significant across periods (*p* < 0.001). Oral diseases and oral pathology retained their importance in both early and late stages of oral health. Furthermore, while oral diseases and oral pathology were more frequently inquired about in the spring, advanced imaging techniques, radiation physics, and temporomandibular joint disorders were also included in the autumn surveys.

**Conclusion:**

ChatGPT-5.2 demonstrated the highest and most consistent accuracy among the evaluated models in DSE oral and maxillofacial radiology questions. In the field of oral and maxillofacial radiology, these model prototypes have the potential to generate new knowledge. Interest in oral diseases and pathology has remained important, while attention to jaw lesions and advanced imaging techniques has increased in recent years.

## Introduction

Dentistry in lasts five years Türkiye. The curriculum includes both basic and clinical sciences. Theoretical knowledge and practical application are provided together. The aim of the program is to enable students to acquire the knowledge, skills, and basic competencies included in the national dental education curriculum [1]. After completing undergraduate education, graduates who want to receive specialist training must take the Dentistry Specialization Examination (DSE) [2]. With the recognition of oral pathology as the latest specialty in Türkiye, Dentistry now has nine main specialties, and oral and maxillofacial radiology is one of them [3, 4].

In parallel with the increasing number of dental faculties in Türkiye, the number of graduates sitting the DSE examination has also risen. As a result, the examination has become increasingly competitive in recent years [5].

Alongside these developments, growing attention has been directed toward artificial intelligence (AI) systems capable of generating human-like responses efficiently and at scale. Various platforms, including ChatGPT, Perplexity AI, YouChat, ChatSonic, Google’s Bard, and Microsoft’s Bing Assistant, have been introduced and rapidly adopted in different fields [6].

Within dentistry, ChatGPT, a conversational AI application developed by OpenAI and released to the public in November 2022, has attracted particular interest as a potential support tool in clinical decision-making and diagnostic processes [7]. Although its clinical use remains under evaluation, several studies have begun to explore its capabilities and limitations [8–11]. The evaluation of the performance of AI like, such as Google Gemini, Microsoft Copilot, and Perplexity, in various areas of dentistry is a current topic. [12–16].

In the literature, Tassoker (2025) [15] and Peker et al. (2025) [16] evaluated the response performance of different AI systems using questions from the 2012–2021 period in the field of oral and maxillofacial radiology.

This study differs from these previous studies in that it includes all the questions and compares a larger number of more current LLM based AI systems. Furthermore, a detailed subject analysis was conducted, examining the data across years and periods.

Accordingly, the aim of this study was to comparatively analyze the answer accuracy of ChatGPT-5.2, ChatGPT-4.0, Gemini, Microsoft Copilot, and Perplexity using oral and maxillofacial radiology specialty examination questions published by the Measurement, Selection and Placement Center of Türkiye between 2012 and 2025.

## Materials and methods

### Ethical approval

This study was conducted exclusively using publicly available online resources and did not involve human participants or animal data. Therefore, ethical committee approval and informed consent were not required.

### Data source and question selection

The questions analysed in this study belong to the DSE oral and maxillofacial radiology branch, conducted between April 2012 and 2025.The total of 200 questions comprising the study material were obtained from a licensed and authorized exam database (DUSDATA), which provides access to officially published DSE questions from the relevant years. Written permission was obtained from DUSDATA. The dataset consists only of questions developed and officially published by the national examination authority. No additional questionnaire was created for this study. Because the questions were prepared by the official institution, their full texts are not included in this article or in the supplementary materials.

### Question characteristics and ınclusion criteria

In the DSE, each examination includes 10 multiple-choice questions in the field of oral and maxillofacial radiology, presented in a five-option, single–best-answer format. No classification was applied based on question format. Rather, all questions were entered into the LLMs based AI systems systems in their original, unmodified form.

### Periodic analysis of question content

In the present study, questions in the domain of oral and maxillofacial radiology from 2012 to 2025 were divided into two time periods, namely the early period (2012–2018) and the late period (2019–2025), with a view to evaluating thematic and structural changes that may occur in the exam content over the years. The results were further analyzed by distinguishing between the spring and fall examination sessions.

### Topic-based content analysis

Each question was assigned to one predefined main topic based on its primary thematic focus. The DUSDATA reference book served as a framework during the categorization process. This helped ensure a consistent approach. To reduce the risk of misclassification, all categories were reviewed twice by the same specialist.

Classification was performed according to the following categories:

1. Oral diseases and oral pathology
2. Anatomical structures
3. Artifacts and imaging errors
4. Radiolucent and radiopaque jaw lesions
5. Caries radiology
6. Dental anomalies
7. Extraoral imaging techniques
8. Imaging
9. Advanced imaging techniques
10. Implant radiology
11. Intraoral radiographic techniques
12. Conventional radiographic techniques and image characteristics
13. Radiation physics and basic principles
14. Clinical and radiographic approach to diagnosis
15. Temporomandibular joint disorders
16. Soft tissue calcifications and ossifications

### Artificial intelligence models

The following AI models were used in this study.

1. ChatGPT-5.2 (OpenAI, San Francisco, CA, USA)
2. ChatGPT-4.0 (OpenAI, San Francisco, CA, USA)
3. Gemini-3 (Google DeepMind, Mountain View, CA, USA)
4. Perplexity AI (Perplexity AI Inc., San Francisco, CA, USA)
5. Claude 4.5 (Anthropic, San Francisco, CA, USA)
6. Microsoft Copilot (Microsoft Corporation, Redmond, WA, USA)

ChatGPT models were accessed via a subscription-based version, while Gemini-3, Perplexity AI, Claude 4.5, and Microsoft Copilot were accessed via their free versions.

### Response generation process using artificial intelligence models

The questions obtained from the Dental Specialty Examination (DSE) were presented to the artificial intelligence models in their original examination format, consisting solely of the question stem and answer options. All questions were sent directly to the AI on January 15, 2026, without any modifications, by a single experienced researcher. Perplexity AI’s free version allowed only a limited number of queries, and eight questions could not be entered. These were excluded from the evaluation.

Consequently, inter-model comparisons were carried out on 192 questions.

### Evaluation of responses

The responses generated by the AI systems were evaluated based on the official Dental Specialty Examination (DSE) answer key published by the Measurement, Selection and Placement Center of Türkiye. Answers were first classified as correct (“C”) or incorrect (“IC”).

Accuracy was subsequently calculated as the percentage of correct responses out of the total questions included in the analysis.

### Statistical analysis

Given that the variables were binary (Correct/incorrect) in nature, it was necessary to employ methods suitable for categorical data, as opposed to making parametric assumptions. Differences in model performance were evaluated using Cochran’s Q test. Following the identification of a substantial discrepancy in Cochran’s Q test, a series of paired comparisons were conducted utilising the McNemar test, with the Bonferroni correction being implemented to account for the multiple tests undertaken. The performance of each model was summarized using descriptive statistics, including frequencies (n) and percentages (%). Data analysis was conducted in SPSS software (IBM Corp., 2017). A p-value of less than 0.05 was considered statistically significant.

## Results

In this present study, a total of 200 questions asked in the field of oral and maxillofacial radiology during the DSE exams between 2012 and 2025 were analysed.

In the 2012–2018 period, ChatGPT-5.2 demonstrated the highest accuracy rate (91.9%), followed by ChatGPT-4.0 (79.8%) and Gemini-3 (70.7%). The remaining models showed substantially lower accuracy rates (Table 1). In the 2019–2025 period, ChatGPT-5.2 again achieved the highest accuracy (95.7%), followed by ChatGPT-4.0 (82.8%) and Gemini-3 (80.6%). The detailed accuracy results of the other models are shown in Table 1. The Cochran Q test revealed a statistically significant difference among the models in both periods (*p* < 0.001).

**Table 1:** Accuracy rates and pairwise comparison of ai systems (2012–2018 vs. 2019–2025)

Years	Types	Accuracy Number	Accuracy Rate	p^a^ value
2012-2018	ChatGPT5.2	91/99	%91,9	**<0.001**
	ChatGPT4.0	79/99	%79,8	
	Gemini-3	70/99	%70,7	
	Perplexity AI	20/99	%20,2	
	Claude 4.5	20/99	%20,2	
	Microsoft Copilot	21/99	%21,2	
2019-2025	ChatGPT5.2	89/93	%95,7	**<0.001**
	ChatGPT4.0	77/93	%82,8	
	Gemini-3	75/93	%80,6	
	Perplexity AI	14/93	%15,1	
	Claude 4.5	19/93	%20,4	
	Microsoft Copilot	18/93	%19,4	

	Pairwise comparison	χ²		p^b^ value
2012-2018	ChatGPT5.2 – ChatGPT4.0	25.384		**0.002**
	ChatGPT5.2 – Gemini-3	0.081		**<0.001**
	ChatGPT5.2 – Perplexity AI	2.203		**<0.001**
	ChatGPT5.2 – Claude 4.5	2.251		**<0.001**
	ChatGPT5.2 – Microsoft Copilot	2.251		**<0.001**
	ChatGPT4.0 – Gemini-3	7.864		0.087
	ChatGPT4.0 – Perplexity AI	3.405		**<0.001**
	ChatGPT4.0 – Claude 4.5	5.734		**<0.001**
	ChatGPT4.0 – Microsoft Copilot	9.068		**<0.001**
	Gemini-3 – Perplexity AI	37.552		**<0.001**
	Gemini-3 – Claude 4.5	63.467		**<0.001**
	Gemini-3 – Microsoft Copilot	63.467		**<0.001**
	Perplexity AI – Claude 4.5	55.594		>0.999
	Perplexity AI – Microsoft Copilot	61.020		>0.999
	Claude 4.5 – Microsoft Copilot	90.134		>0.999
2019-2025	ChatGPT5.2 – ChatGPT4.0	25.858		**0.003**
	ChatGPT5.2 – Gemini-3	4.224		**0.007**
	ChatGPT5.2 – Perplexity AI	0.741		**<0.001**
	ChatGPT5.2 – Claude 4.5	0.113		**<0.001**
	ChatGPT5.2– Microsoft Copilot	0.264		**<0.001**
	ChatGPT4.0 – Gemini-3	46.314		0.754
	ChatGPT4.0 – Perplexity AI	34.019		**<0.001**
	ChatGPT4.0 – Claude 4.5	26.518		**<0.001**
	ChatGPT4.0 – Microsoft Copilot	28.892		**<0.001**
	Gemini-3 – Perplexity AI	46.497		**<0.001**
	Gemini-3 – Claude 4.5	70.176		**<0.001**
	Gemini-3 – Microsoft Copilot	75.232		**<0.001**
	Perplexity AI – Claude 4.5	34.272		0.227
	Perplexity AI – Microsoft Copilot	46.497		0.289
	Claude 4.5 – Microsoft Copilot	69.215		>0.999

*p *<0.05 significant value, ^a^Coachran Q test, ^b^Mc-Nemar test

Bold values indicate statistically significant differences(*p *< 0.05)

The McNemar test results indicated that ChatGPT-5.2 showed a statistically significant difference in accuracy when compared with ChatGPT-4.0, Gemini-3, Perplexity AI, Claude 4.5, and Microsoft Copilot in both periods (*p* < 0.05). No statistically significant difference was observed between ChatGPT-4.0 and Gemini-3 (*p* > 0.05). However, ChatGPT-4.0 and Gemini-3 both showed statistically significant differences compared with Perplexity AI, Claude 4.5, and Microsoft Copilot (*p* < 0.001). In contrast, no statistically significant difference was found among Perplexity AI, Claude 4.5, and Microsoft Copilot (*p* > 0.05) in pairwise comparisons (Table 1).

According to Table 2, in the 2012–2018 period, the highest proportion of questions was related to oral diseases and oral pathology (23.5%). Radiolucent and radiopaque jaw lesions were the second most common category (19.6%). Conventional radiographic techniques, image characteristics, and radiation physics/basic principles followed this category. Advanced imaging techniques made up a smaller proportion of the questions. During the 2019–2025 period, oral diseases and oral pathology (20.4%) and radiolucent and radiopaque jaw lesions (21.4%) again represented the largest share of questions. Advanced imaging techniques and extraoral imaging techniques each accounted for 10.2%, whereas imaging and temporomandibular joint disorders appeared less frequently.

**Table 2:** Distribution of topic headings in two distinct periods (2012–2018 and 2019–2025)

Distribution of Topics	2012–2018 (*n*=102)	2019–2025 (*n*=98)
Oral Diseases and Oral Pathology	24	20
Anatomical Structures	4	5
Artifacts and Imaging Errors	2	0
Radiolucent and Radiopaque Lesions of the Jaw	20	21
Caries Radiology	1	1
Dental Anomalies	4	1
Extraoral Imaging Techniques	5	10
Imaging	0	5
Advanced Imaging Methods	9	10
Implant Radiology	1	0
Intraoral Radiographic Techniques	0	1
Conventional Radiographic Techniques and Image Characteristics	13	1
Radiation Physics and Basic Principles	11	13
Clinical and Radiographic Approach to Diagnosis	0	1
Temporomandibular Joint Disorders	0	3
Soft Tissue Calcifications and Ossifications	8	6

In the 2012–2018 spring sessions, oral diseases and oral pathology (30.0%) and radiolucent and radiopaque jaw lesions (20.0%) were the most frequent topics, followed by conventional radiographic techniques and image characteristics and radiation physics and basic principles (each 20.0%). Other topics were represented at lower rates. In the 2012–2018 fall sessions, Oral diseases and oral pathology (20.8%) and radiolucent and radiopaque jaw lesions (19.4%) were again the most common, followed by advanced ımaging techniques (11.1%), radiation physics and basic principles (9.7%), and soft tissue calcifications and ossifications (8.3%) (Table 3).

**Table 3:** Distribution of topics by spring and fall sessions across two periods

Topic heading	2012–2018 Spring (*n*=30)	2012–2018 Fall (*n*=72)	2019–2025 Spring (*n*=39)	2019–2025 Fall (*n*=49)
Oral Diseases and Oral Pathology	9	15	10	10
Anatomical Structures	1	3	1	4
Artifacts and Imaging Errors	0	2	0	0
Radiolucent and Radiopaque Lesions of the Jaw	6	14	9	12
Caries Radiology	1	0	0	1
Dental Anomalies	0	4	1	0
Extraoral Imaging Techniques	0	5	4	6
Imaging	0	0	2	3
Advanced Imaging Methods	1	8	3	7
Implant Radiology	0	1	0	0
Intraoral Radiographic Techniques	0	0	1	0
Conventional Radiographic Techniques and Image Characteristics	6	7	0	1
Radiation Physics and Basic Principles	6	7	4	7
Clinical and Radiographic Approach to Diagnosis	0	0	1	0
Temporomandibular Joint Disorders	0	0	0	3
Soft Tissue Calcifications and Ossifications	0	6	3	5

In the 2019–2025 spring sessions, oral diseases and oral pathology (25.6%) and radiolucent and radiopaque jaw lesions (23.1%) had the highest proportions, while extraoral ımaging techniques (10.3%), radiation physics and basic principles (10.3%), and advanced imaging techniques (7.7%) were also represented. In the 2019–2025 fall sessions, radiolucent and radiopaque jaw lesions accounted for 24.5% and oral diseases and oral pathology for 20.4%. Questions were also asked on extraoral imaging techniques (12.2%), advanced imaging methods (14.3%), and radiation physics (14.3%). Soft tissue calcifications and ossification accounted for 10.2%, while temporomandibular joint disorders comprised 6.1% (Table 3).

## Discussion

To the best of our knowledge, no previous study in oral and maxillofacial radiology has comprehensively incorporated the entire set of examination questions, analysed their topic distribution according to years and examination periods, and comparatively assessed multiple up to date large language models within a unified analytical design.

In the study, ChatGPT-5.2 (91.9%-95.7%) and ChatGPT-4.0 (70.7%-82.8%) showed the highest accuracy rates on DSE oral and maxillofacial radiology questions in both the early and late stages. In both periods, ChatGPT-5.2 demonstrated the highest level of accuracy; conversely, Perplexity AI (20.2%-15.1%), Claude 4.5 (20.1%-20.4%), and Microsoft Copilot (21.2%-19.4%) exhibited lower levels of accuracy. In a previous study, Tassoker et al.¹^5^ reported that ChatGPT-4.0 Omni achieved a higher accuracy rate (86.1%) than other models. Within this category, ChatGPT-4 Omni achieved the highest number of correct responses. Similarly, Peker et al.¹^6^ reported that ChatGPT-4.0 (91.1%) and Microsoft Copilot (86.2%) exhibited comparably high performance levels. Although AI models differ in studies on oral and maxillofacial radiology in DSE in the literature, the results are largely consistent. On the other hand, the fact that this study was conducted more recently and used current models may explain the higher accuracy rates of this study, especially for ChatGPT. In addition, other studies did not differentiate between temporal periods, early/late periods, or autumn and spring, so a direct comparison is not possible in this sense. Additionally, in a study conducted outside of exam-focused assessments in the literature regarding disease knowledge, ChatGPT, Perplexity, and Gemini were evaluated in terms of readability, information quality, and reliability of their responses to user questions about ankylosing spondylitis. The study reported that Perplexity scored relatively higher in these criteria [17]. In this context, models that received low scores in question-focused studies may also have potential in terms of medical information.

Studies outside the oral and maxillofacial radiology fields are also available in the literature. For example, in a study conducted in the field of endodontics, Çekiç reported that there was no significant difference in overall accuracy between ChatGPT-4.0, Gemini Advanced, Microsoft Copilot and Claude, but Microsoft Copilot and Claude showed relatively higher accuracy in previous years’ exam questions [18]. In a study conducted by Haberal et al. in the field of restorative dentistry, the performance of eight different artificial intelligence models was evaluated using questions from the 2012–2025 Dental Specialty Examination (DSE). Although no statistically significant difference was found between the models in terms of overall accuracy rates, the study reported that Gemini Advanced and ChatGPT-4.0 Plus had relatively higher accuracy rates [19]. Furthermore, studies in the disciplines of pedodontics and prosthetic dentistry have shown that the performance of LLMs can vary depending on the specific discipline. While Aşık et al. [20] reported that ChatGPT-3.5 showed moderate accuracy in DSE questions in the field of pedodontics and that this accuracy changed over time, Bilgin et al. [21] showed that the performance of ChatGPT-3.5 and Gemini was limited in prosthetic dentistry questions and that there was no serious inconsistency between the models. Moreover, Şişmanoğlu et al. reported that ChatGPT-4.0 and Gemini Advanced were able to exceed the DSE passing threshold; however, their performance had not yet reached the level of human candidates who received the highest scores [22]. In a study by Çetiner et al. in the field of oral and maxillofacial surgery, ChatGPT-5 (93.0%) and Gemini 2.5 Pro (96.9%) showed high accuracy rates. However, despite the numerical difference, it was reported that there was no statistically significant difference between the models [23].

An analysis of the subject distribution of the exam questions reveals that some topics were represented at a higher rate in both periods. In both the early and late periods, the questions were predominantly focused on oral diseases and oral pathology, and radiolucent-radiopaque jaw lesions.

On the other hand, when the subject distributions are analyzed according to the fall and spring sessions differences in content are observed. While the spring semesters most frequently focus on topics such as oral diseases and oral pathology, radiological lesions, and radiation physics, the fall sessions while maintaining the prominence of oral diseases and oral pathology and radiological lesions, also include more technical and clinically specific topics such as advanced imaging techniques and temporomandibular joint disorders. This trend is also confirmed by similar content analyses in the existing literature. Yalçın et al. reported that for the period 2012–2025, the highest percentages of DSE questions were concentrated in the topics of radiological pathologies and imaging techniques. On the other hand, although the subject distribution was analyzed by year in the same study, it was not separated according to fall, spring, early and late periods [24]. In the study conducted by Gerzeli et al. [25] in the field of oral and maxillofacial radiology, a total of 182 questions from 19 DSE sessions held between 2012 and 2025 were analysed. When the distribution of questions was examined, oral diseases and their interpretation were the most frequent, followed by imaging methods and basic radiology. There were no questions from forensic dentistry, and one question was asked about implants. In a separate investigation by Ekici et al. [26], a total of 128 questions in the field of oral and maxillofacial surgery were evaluated according to years, and it was found that the highest percentage of questions were related to oral diseases and the lowest percentage were related to orofacial pain.

Several limitations should be considered when evaluating the study’s results. First, questions were classified only according to subject headings, and no analysis of question types was conducted. Second, the continuous updating of LLMs and the limitation of the research to a single field of expertise are among the other limitations of the study. Furthermore, the fact that AI models are constantly updated and that the study data was collected within a specific timeframe, as well as whether the models are subscription-based, should be considered when interpreting the results. On the other hand, studies conducted with previously accessible questions may have influenced the high accuracy rates of some models. Finally, it should be noted that the study compared 192 questions using a free version of an AI model (Perplexity AI).

In future studies, it may be better to classify the questions not only by subject, but also by their difficulty level, format, and case complexity. This may help compare the performance of AI models more clearly across different types of content.

## Conclusion

Among the models analysed in the study, ChatGPT-5.2 attained both the highest and most stable accuracy rate. In the field of oral and maxillofacial radiology, these model prototypes have the potential to generate new knowledge. Although the weight of oral diseases and oral pathology was maintained in the basic subject distribution, the increase in the share of jaw lesions and advanced imaging content in the exam in recent years is noteworthy.

## Data Availability

The data supporting the findings of this study are available from the corresponding author upon reasonable request.

## Abbreviations

AI: Artificial Intelligence
DSE: Dental Specialty Examination
DUSDATA: A private educational institution providing preparatory materials for the DUS examination
LLMs: Large Language Models
